# Radon and Thoron; Radioactive Gases Lurking in Earthen Houses in Rural Kenya

**DOI:** 10.3389/fpubh.2019.00113

**Published:** 2019-05-08

**Authors:** Margaret Chege, Nadir Hashim, Catherine Nyambura, Amidu Mustapha, Masahiro Hosada, Shinji Tokonami

**Affiliations:** ^1^Physics Department, Kenyatta University, Nairobi, Kenya; ^2^Physics Department, Federal University of Agriculture Abeokuta, Abeokuta, Nigeria; ^3^Department of Radiological Life Sciences, Hirosaki University Graduate School of Health Sciences, Hirosaki, Japan; ^4^Department of Radiation Physics, Institute of Radiation Emergency Medicine, Hirosaki University, Hirosaki, Japan

**Keywords:** radon, thoron, lung cancer, mud-walled, traditional dwellings

## Abstract

In this paper, documented studies on radon and thoron concentrations in earthen dwellings and ^238^U and ^232^Th concentrations in soil in Kenya are reviewed. High concentrations of the isotopes were recorded in the earthen dwellings despite being generally well ventilated. Mrima Hill in the Coast region recorded the highest thoron levels with a mean of 652 Bq m^−3^. Twenty five percent of dwellings had thoron concentration in excess of 1,000 Bq m^−3^. Notably high indoor radon levels were recorded in Taita Taveta also in the Coast region, and in Kenyatta University situated in Nairobi in the Central region of the country. Radon concentration in the Rift Valley region was found to be too low to contribute significantly to radiation exposure. Based on studies on the concentration of ^238^U and ^232^Th in soil, the Southwestern region of the country was anticipated to have elevated radon/thoron concentrations in earthen dwellings. Existing studies involving measurement of indoor radon and thoron, and ^226^Ra and ^232^Th in soil are relatively few and of a small scale. More extensive studies are therefore necessary not only to corroborate the risk projections but to also generate sufficient data to enable countrywide mapping of indoor radon/thoron risk-prone areas.

## Introduction

Radon and thoron gases can become airborne under favorable conditions and accumulate in confined spaces such as dwellings thus becoming significant sources of internal radiation exposure (1). While the importance of indoor radon to lung cancer (2) is well documented, that of indoor thoron is largely under-represented. The main source of the isotopes in (modern) houses is soil-gas seepage through building foundation and with a half-life of 55.6 s, thoron atoms can largely decay before exiting the foundation. The fraction that eventually becomes airborne is often considered too low to contribute significantly to radiation exposure. This however is not necessarily true. This is because the risk resulting from radon/thoron exposure depends, among other factors, on the activity of the radionuclides and from a theoretical perspective, a considerably small fraction of thoron atoms is required to produce the same activity as radon atoms. As an example, it requires just 17 thoron atoms to produce the same activity as 100,000 radon atoms due to the different half-lives. Even with equal activity, risk resulting from thoron exposure is higher as thoron progeny are cumulatively airborne for a longer period of time. According to UNSCEAR (3), the risk from thoron progeny based on its long-lived progeny ^212^Pb is about 4 times that of an equal concentration of radon progeny. It is worth noting that use of soil as a building material is a common practice in Kenya. Thoron exhalation rate from soil can be many times higher than that of radon (4, 5) hence the likelihood of elevated thoron concentrations in earthen dwellings. This has been observed in cave dwellings in China (6). Studies are continually emerging linking relatively low thoron levels to notable radiation exposure. In Germany (7) for instance thoron concentration up to 90 Bq m^−3^ was associated with inhaled dose up to 4 mSv y^−1^ which is nearly half the upper bench mark for effective inhaled dose (8).

The ICRP in its latest publication proposes new dose conversion coefficients for radon progeny (9) which have been found to double the risk from the same as before radon concentration (10, 11). This raises not only the toxicity index of radon but also of thoron. The contribution of airborne thoron alongside that of radon particularly in earthen dwellings can therefore not be overlooked. In Kenya, a significant proportion of the rural population resides in earthen dwellings. Though anticipated, effects of exposure such as lung cancer are hard to ascertain due to insufficient cancer screening equipment in rural-based health facilities. In some instances, lung cancer is mistakenly diagnosed and managed as pulmonary tuberculosis (TB) (12), one of the leading causes of death in the country (13). This paper reviews the documented studies on radon and thoron concentrations in earthen dwellings in Kenya, in addition to measurement ^238^U and ^232^Th in soil. This is aimed at generating information on areas with a high risk of indoor radiation exposure. The information provided in this report can be used as a basis for countrywide research on the radionuclides in different geographical and geological areas which is important for mapping risk areas, determining radon and thoron reference levels and also in setting up of lung cancer screening centers.

## Materials and Methods

### Earthen Dwellings in Rural Kenya

House design in rural parts of the country has barely changed over the years save for a few differences. Currently, most dwellings bear the rectangular shape as opposed to circular one as this allows for multiple rooms to be incorporated. Dwellings are normally put up in family or individual-owned parcels of land and generally, no building regulations or codes are followed during construction. Site for a new dwelling is usually leveled, a double wall-mesh made using long wooden poles erected, and mud manually packed in the space between the wall-mesh. The dwellings don't usually contain a ceiling beneath the thatched roof which allows for increased air circulation. Nonetheless, rural dwellers prefer staying outdoors even when not working and as such indoor occupancy is often approximated as between 50 and 60%.

### Sampling Regions

**Figure 1:**
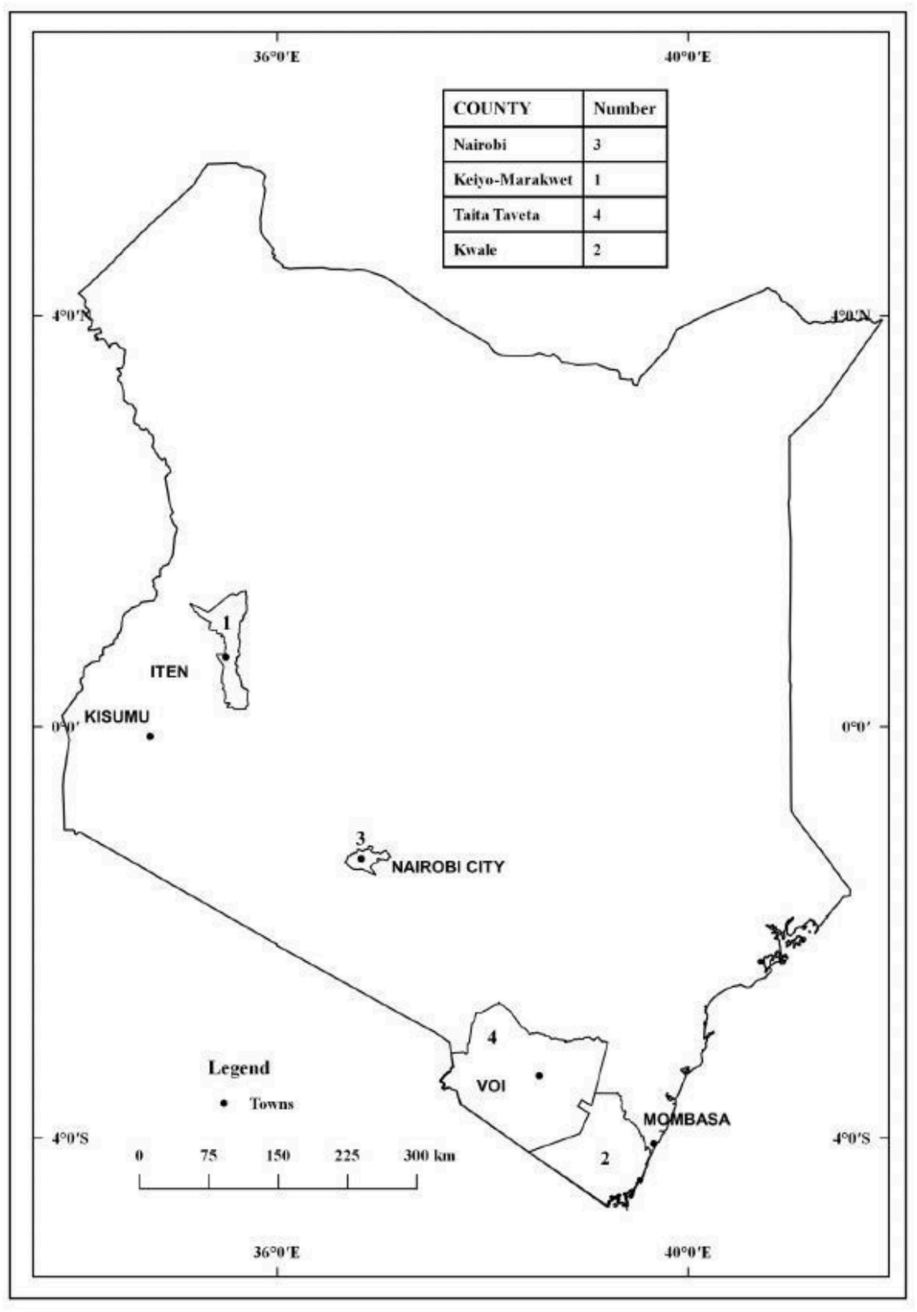
Map of Kenya showing the sampling regions.

Figure 1 shows the regions where the reviewed studies were carried out. The sampling areas fall within three administrative regions namely the Coast (14–16), Nairobi (Central) (17, 18) and Rift Valley (14). The Coast study was performed in two regions: Taita Taveta County (14) and Mrima Hill (15, 16). Taita Taveta is located about 150 km northwest of the port city of Mombasa and about 350 km southeast of the capital city Nairobi. According to the 2009 census, the region has a population of about 284,000 (of about 40 million total population). It is fairly cool with mean temperature of about 23°C. Mrima Hill on the other hand is located in Kwale County in the southern region of the country and lies at about 65 km southwest of the port city of Mombasa. Mrima Hill region straddles Kinango and Msambweni sub-counties with a combined population of about 333,000. Mrima Hill region is often hot and humid with temperatures averaging 26°C. The Nairobi study (17, 18) was carried out in Kenyatta University located in Kahawa region off the Thika superhighway and at approximately 20 km from Nairobi Central Business District (CBD). Kahawa region extends into Ruiru sub-county in Central Kenya and has a population of about 200,000. The Kahawa region has a mild warm and temperate climate and average temperature of about 20°C. The Rift Valley study was carried out in Soi. Soi is located in formerly Keiyo District (now part of Elgeyo Marakwet County) in the Kerio Valley. Keiyo has a population of about 143,000.

In terms of geology, Taita Taveta is characterized by metamorphic rocks in the Mozambique belt and the Tertiary Volcanic belt. Taita region is associated with the former and Taveta with the latter (19, 20). The region is endowed with an array of mineral deposits and gemstones albeit largely un-exploited. These include red, green and yellow garnets, blue and pink sapphire among others. Mrima Hill region on the other hand is constituted by corals, sandy hills consisting of Magarini sands, and Jurassic carbonatite rock formation. The region has rich mineral deposits such as iron, manganese and niobium. Nairobi area is underlain by tertiary volcanics characterized by different types of clays (21) similar to most parts of Central Kenya, while Soi is characterized by alluvial plain underlain by gneisses. Soi region has mineral deposits such as fluorspar.

### Methods

The methods used in the various studies included electret ionization chambers, CR39 solid state nuclear tract detectors (SSND) and activated charcoal. The electret technique consisted of an ionization chamber fitted with an active electret. After obtaining the initial electret voltage, the detector system was set up in the houses of interest for 2–30 days. After exposure, the drop on voltage was determined and correlated to radon concentration. In the activated charcoal technique, about 50 g of activated charcoal contained in metal canisters were used. The canisters were deployed in dwellings of interest for a period of 48 h before being sealed and left to sit for about 3 h to allow for the decay of thoron. Analysis was then done using Na I(Tl) detector and radon concentration quantified based on ^214^Bi gamma line. Weekly measurements were carried out for a period of 3 months. Pairs of CR39 SSND detectors were used to simultaneously measure radon and thoron concentrations. To achieve this, one detector was put in a canister that retarded thoron entry such that it decayed without accessing the detector. The difference in track densities between the twin detectors was attributed to thoron. In the field, the twin detector detectors were set up side by side in dwelling of interest at a distance of about 20 cm from the wall. They were left in place for a period of 3 months. After exposure the detectors were etched and tracks density used to quantify radon and thoron concentrations.

## Results and Discussion

### Radon and Thoron Research in Traditional Dwellings

Table 1 summarizes the values of radon and thoron measurements carried out in the three regions. Significant levels of radon were observed in Taita Taveta region with nearly a quarter of the dwellings registering concentrations in excess of 400 Bq m^−3^. The maximum concentration was about twice the ICRP upper reference level of 300 Bq m^−3^. While the elevated radon levels were attributed to reduced ventilation, high radon exhalation rate and possibly elevated content of ^226^Ra in the building materials cannot be ruled out. The documented results do not reflect the contribution of thoron. This means that the inhaled dose may be higher than reported.

**Table 1:** Summary statistics of radon and thoron concentrations.

**Region**	**Sub-region** **(number of dwellings)**	**Concentration (Bq m**^****−3****^**)** **mean (range)**	
		**Radon**	**Thoron**
Coast	Taita Taveta^*^ (14) (54 dwellings)	259 (43–407)	–
	Mrima Hill^**^ (15, 16) (20 dwellings)	35 (16–56)	652 (132–1,295)
Nairobi (central)	Kenyatta University^***^ (17, 18) (4 dwellings)	170 (30–315)	–
Rift valley	Soi^*^ (14) (42 dwellings)	67 (14–161)	–

*Measurement techniques*:

* *electret technique*;

** *CR39 SSNT detectors*,

*** *activated charcoal*.

**Figure 2:**
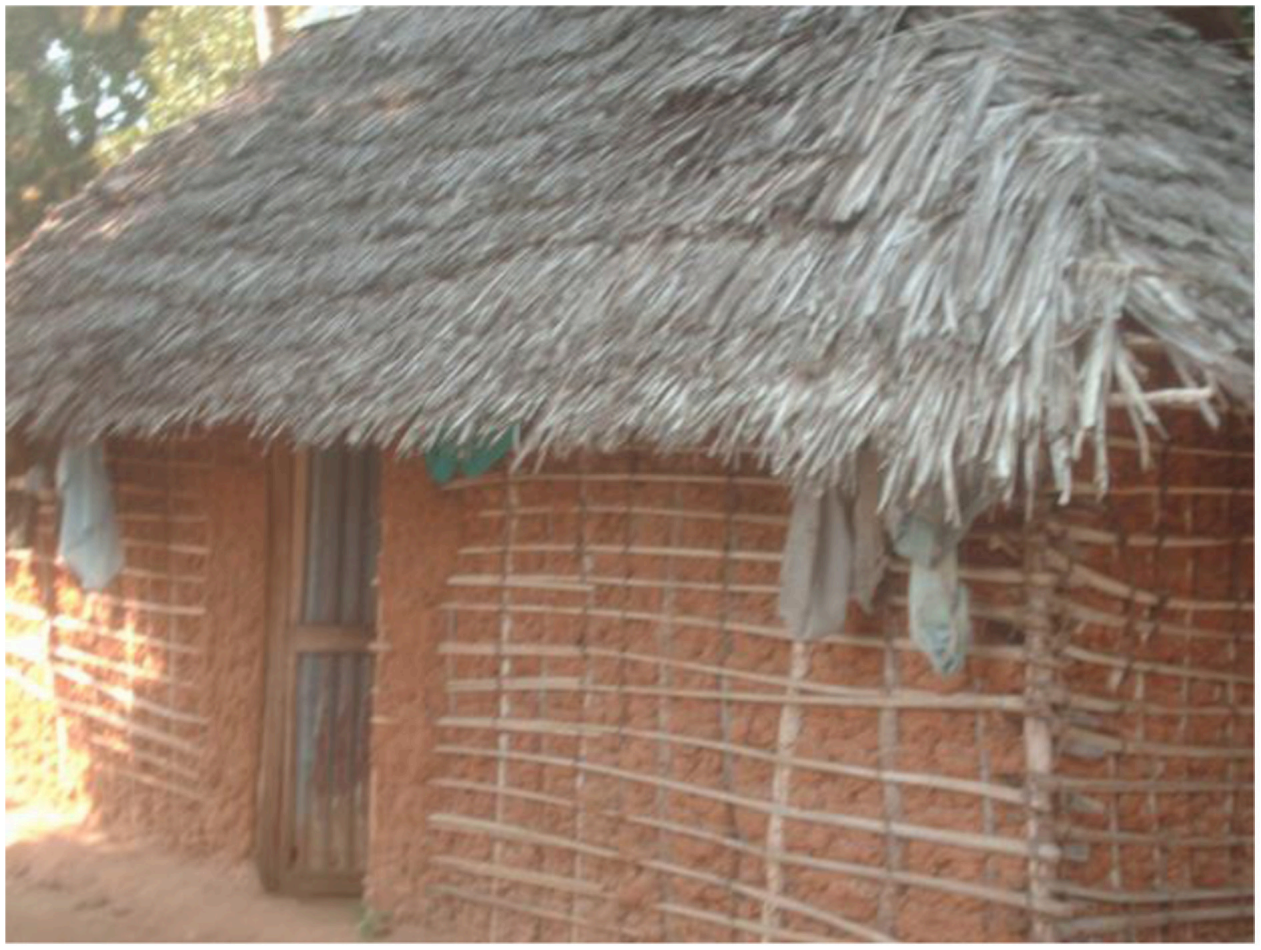
A dwelling in Mrima Hill region.

Mrima Hill was the first region in the country to be declared a high background radiation area after high outdoor radiation dose of up to 106 mSv y^−1^ was discovered (22) in 1991. Twenty years later, analysis of soil samples (23) revealed high concentrations of ^232^Th with a mean of 500 Bq kg^−1^ while that of ^238^U averaged 207 Bq kg^−1^. That very soil was used in the construction of dwellings in which radon and thoron measurements were carried out. Figure 2 shows a typical dwelling found in Mrima Hill region. The region recorded significantly high indoor thoron concentrations averaging 652 Bq m^−3^. A quarter of the dwellings had thoron concentration exceeding 1,000 Bq m^−3^. The magnitude of the thoron problem in Mrima hill region may be best understood if looked at from radon perspective. Assuming a tentative conversion factor of four, the radon-equivalent of thoron mean concentration becomes 2,608 Bq m^−3^ which is nearly nine times the ICRP maximum reference level. The high thoron concentration was attributed to high content of ^232^Th in the building materials as well as high thoron exhalation rate which averaged 19.6 Bq m^−2^ s^−1^. The fact that the dwellings had fairly good ventilation did not appear to inhibit thoron build-up. Radon concentration was markedly low, averaging 35 Bq m^−3^. Its exhalation rate was nearly 5,000 times lower than that of thoron.

**Figure 3:**
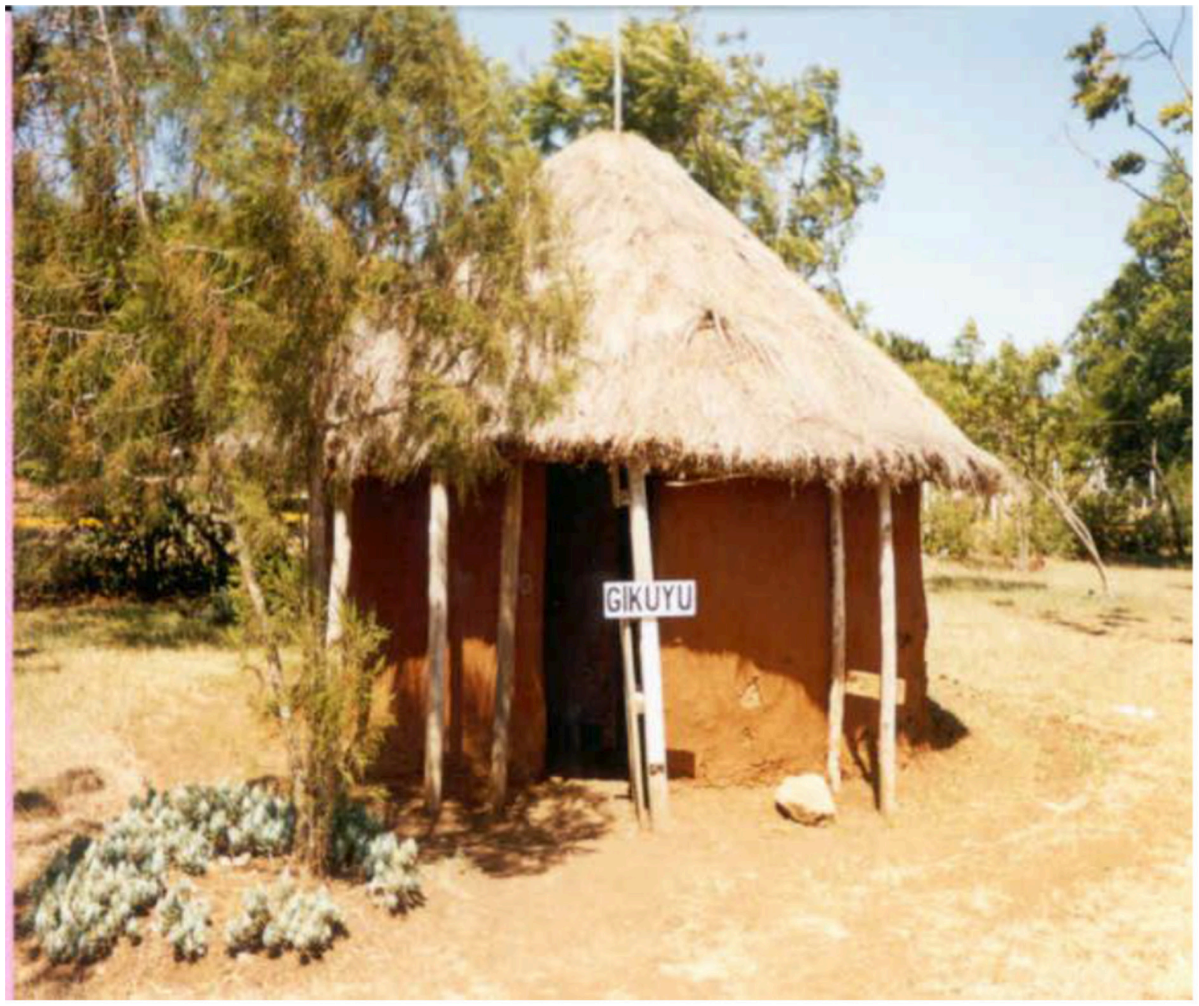
Model traditional dwelling in Kenyatta University.

One of the model huts used for the Nairobi study is shown in Figure 3. The huts studied were single roomed and un-inhabited, and the doors and windows remained open the entire sampling period (day and night). Radon concentration averaged 170 Bq m^−3^ with a maximum of 315 Bq m^−3^. This was considered rather high given the abnormally high ventilation. The relatively high radon concentration was attributed to high porosity of the building materials coupled with elevated concentration of ^226^Ra which averaged 101 Bq kg^−1^, nearly three-fold the world average of 35 Bq kg^−1^. Further analysis of the building materials revealed ^232^Th concentration about five times the world average of 30 Bq kg^−1^. This implies that if non-discriminative technique was used, and if doors and windows were shut at night as happens in habited dwellings, the radiation levels in the model huts would be much higher than reported. Elevated concentrations of ^232^Th and ^226^Ra in soil have been reported in other areas in the outskirts of Nairobi city. In Mwiki and Industrial Area (24) for instance, ^232^Th concentration in soil averaged 198 Bq kg^−1^ and 228 Bq kg^−1^ respectively while that ^226^Ra was 96 Bq kg^−1^ and 159 Bq kg^−1^ respectively. This is crucial in that the two areas have informal settlements consisting of metal-walled, earthen-floored dwellings. With high ^232^Th and ^226^Ra concentration in soil therefore, floors of such dwellings can be important sources of indoor thoron and radon.

In the Soi region notably low indoor radon concentrations with a mean value of 67 Bq m^−3^ and 161 Bq m^−3^ respectively were reported. The relatively low radon levels were attributed to high ventilation occasioned by high temperature often witnessed in the region. Surprisingly, the study found radon levels in modern stone houses higher by a factor of three compared to the earthen ones. The high radon levels were attributed to uranium-bearing minerals possibly incorporated in fluorspar deposits in the building stones. In addition, the modern dwellings were found to be more airtight when compared with earthen dwellings. Concentrations of ^226^Ra and ^232^Th in the soil and stone are however not given in the reviewed report. The report does not discuss thoron concentration in either earthen or modern dwelling.

### Potential Risk Areas in Kenya

One area of interest in terms of potential for elevated indoor thoron concentration is the southwestern region of the country. This is home to Homa and Ruri mountains which are considered high background radiation areas. In the Homa Mountains, activity concentrations of ^232^Th and ^226^Ra in soil with means of 409 Bq kg^−1^ and 195 Bq kg^−1^ and highs of 1,447 Bq kg^−1^ and 1,369 Bq kg^−1^ are documented (25). Radioactivity levels were higher in Ruri Mountains (26) with ^232^Th concentration averaging 1,396 Bq kg'1 with a high of 6,559 Bq kg^−1^ and ^238^U averaging 178 Bq kg^−1^ with a maximum of 499 Bq kg^−1^. Clearly, ^232^Th is the main source of radiation exposure in the regions and given that traditional earthen dwellings are a commonplace in the region, thoron is likely to contribute significantly to the total radiation dose.

## Conclusion

This paper reviews thoron and/or radon studies involving collectively 66 earthen dwellings in the Coast, Rift Valley and Nairobi (Central) regions of Kenya. Evidently, in addition to radon, thoron is a potential source of radiation exposure in earthen dwellings typical to rural Kenya. In the high background radiation area of Mrima Hill, a quarter of the dwellings studied had thoron concentration of over 1,000 Bq m^−3^. Taita Taveta region also reported elevated concentrations of indoor radon, as did Nairobi area. For the most part, normal ventilation did not seem to effectively prevent the build-up of the isotopes in indoor air. Additionally, high ^238^U and ^232^Th concentrations in soil were observed in Mwiki and Industrial Area within the larger Nairobi region, as well is in Homa and Ruri Mountains in Southwestern region of the country. Based on reviewed reports on indoor radon/thoron levels and the concentrations of ^238^U and ^232^Th in soils, an increasing order of high risks of radon/thoron exposures is likely to be reported in Coast, Nairobi (Central) and Southwestern regions of the country whereas low risk is expected in the Rift Valley region. However, more representative studies on indoor radon and thoron as well as measurement of ^226^Ra and ^232^Th concentration in soil are required in these regions to corroborate these projections.

## Author Contributions

All authors listed have made a substantial, direct and intellectual contribution to the work, and approved it for publication.

### Conflict of Interest Statement

The authors declare that the research was conducted in the absence of any commercial or financial relationships that could be construed as a potential conflict of interest.

## References

[1] UNSCEAR United Nations Scientific Committee on Effects of Atomic Radiation Report to the General Assembly: Sources and Effects of Ionizing Radiation. New York, NY: United Nations (2008).

[2] Zeeb Hajo Shannoun Ferid WHO World Health Organization Handbook on Indoor Radon: A Public Health Perspective. Geneva, Switzerland World Health Organization (2009). Available online at: http://www.who.int/iris/handle/10665/44149

[3] UNSCEAR United Nations Scientific Committee on Effects of Atomic Radiation Report to the General Assembly: Sources and Effects of Ionizing Radiation. New York, NY: United Nations (2000).

[4] Syarbaini S Pudjadi E Radon and Thoron Exhalation Rates from Surface Soil of Bangka - Belitung Islands, Indonesia. Indonesian J Geosci. (2015) 2:35–42. 10.17014/ijog.2.1.35-42

[5] Hosoda M Sorimachi A Yasuoka Y Ishikawa T Sahoo SK Furukawa M. Simultaneous measurements of radon and thoron exhalation rates and comparison with values calculated by UNSCEAR equation. J Radiat Res. (2009) 50:333–43. 10.1269/jrr.08121 19506347

[6] Tokonami S Sun Q Akiba S Zhuo W Furukawa M Ishikawa T. Radon and thoron exposures for cave residents in Shanxi and Shaanxi provinces. Radiat. Res. (2004) 162:390–6. 10.1667/RR3237 15447044

[7] Gierl S Meisenberg O Feistenauer P Tschiersch J Thoron and thoron progeny measurements in German clay house. Radiat Prot Dosim. (2014) 160:160–3. 10.1093/rpd/ncu076 24743764

[8] ICRP The 2007 Recommendations of the International Commission on Radiological Protection. Ann. ICRP. (2007) ICRP Publication 103, 37:2–4. 10.1016/j.icrp.2007.10.003 18082557

[9] ICRP Occupational Intakes of Radionuclides: Part 3. Ann. ICRP. (2017) ICRP Publication 137 46:3/4. 10.1177/0146645317734963 29380630

[10] Strahlenschutzkommission Radon dose coefficients Recommendation by the German Commission on Radiological Protection. (2018) Bonn, Germany.

[11] Australian Government ARPANSA Advisory Note. New Dose Coefficients for Radon Progeny: Impact on Workers and the Public. Australian Radiation Protection and Nuclear Safety Agency (2018).

[12] Atundo L Chite F Chesumbai G Kosgei S Incidences and trends of lung cancer in Western Kenya for the period between 2012-2016. J Glob Oncol. (2018) 4:201 10.1200/jgo.18.81400

[13] Republic of Kenya, Ministry of Health National Strategic Plan on Tuberculosis, Leprosy and Lung Health 2015-2018. NTLD-Program 2014 (2014).

[14] Maina DM Kinyua AM Nderitu SK Agola JO Mangala MJ Indoor Radon (222Rn) Levels in Coastal and Rift Valley Regions of Kenya. International Atomic Energy Agency (IAEA), IAEA-CN-91/56, 401-405 (2002).

[15] Chege MW Hashim NO Merenga AS Meisenberg O Tschiersch J. Estimation of annual effective dose due to radon and thoron concentrations in mud dwellings of Mrima Hill, Kenya. Radiat Prot Dosim. (2015) 167:139–42. 10.1093/rpd/ncv231 25920792

[16] Chege MW Modelling Radon and Thoron Exhalation and Measurement of Total Natural Radiation Exposure in Mrima Hill, Kenya. PhD Thesis, Kenyatta University (2014).

[17] Chege MW Rathore IVS Chhabra SC Mustapha AO. The influence of meteorological parameters on indoor radon in selected traditional Kenyan dwellings. J Radiol Prot. (2009) 29:95–103. 10.1088/0952-4746/29/1/007 19225187

[18] Chege MW Screening Measurement of Indoor Radon-222 Concentrations by Gamma Ray Spectrometry in Kenyatta University. M.Sc Thesis (Physics) Kenyatta University, Nairobi (2007).

[19] Rop BK Economic and Job Creation Potential of Artisanal and Small-Scale Mining in Taita Taveta County, Kenya. UNDP (2014). Available online at: http://www.jkuat.ac.ke/departments/mining/wp-content/uploads/2017/10/Small-Scale-Mining-n-Taita-Taveta-County-Kenya.pdf (accessed on January 4, 2019).

[20] Taita Taveta County Government: Supporting Quality Life for the People of Taita Taveta. The First Taita Taveta County Integrated Development Plan 2013-2017.

[21] University of Nairobi, Department of Engineering Engineering Properties of Soils Case Study: Nairobi Area. (2018). Available online at: http://geology.uonbi.ac.ke/node/1949 (accessed December 9, 2018).

[22] Patel JP Environmental radiation survey of the area of high natural radioactivity of Mrima Hill of Kenya. Discov Innovat. (1991) 3:31–5.

[23] Kebwaro JM Rathore IVS Hashim NO Mustapha AO Radiometric assessment of natural radioactivity levels around Mrima Hill, Kenya. Int J Phys Sci. (2011) 6:3105–10. 10.5897/IJPS11.052

[24] Chege BW Analysis of Radiation Levels in Nairobi's Central Business District and the Industrial Area, Kenya. MSc Thesis, Kenyatta University (2015).

[25] Otwoma D Patel JP Bartilol S Mustapha AO. Estimation of annual effective dose and radiation hazards due to natural radionuclides in mount homa, southwestern Kenya. Radiat Protect Dosimetry. (2013) 155:497–504. 10.1093/rpd/nct031 23486485

[26] Achola SO Radioactivity and Elemental Analysis of Carbonatite Rocks From Parts of Gwasi Area, South Western Kenya. Master of Science (Physics), University of Nairobi (2009).

